# Impact of particulate air pollution on airway injury and epithelial plasticity; underlying mechanisms

**DOI:** 10.3389/fimmu.2024.1324552

**Published:** 2024-03-08

**Authors:** Özgecan Kayalar, Hadi Rajabi, Nur Konyalilar, Deniz Mortazavi, Gizem Tuşe Aksoy, Jun Wang, Hasan Bayram

**Affiliations:** ^1^ Koç University Research Center for Translational Medicine (KUTTAM), Koç University School of Medicine, Istanbul, Türkiye; ^2^ Department of Biomedicine and Biopharmacology, School of Biological Engineering and Food, Hubei University of Technology, Wuhan, Hubei, China; ^3^ Department of Pulmonary Medicine, School of Medicine, Koç University, Zeytinburnu, Istanbul, Türkiye

**Keywords:** particulate matter (PM), airway inflammation, mucociliary clearance, epithelial barrier integrity, epithelial plasticity, airway remodeling, asthma, COPD

## Abstract

Air pollution plays an important role in the mortality and morbidity of chronic airway diseases, such as asthma and chronic obstructive pulmonary disease (COPD). Particulate matter (PM) is a significant fraction of air pollutants, and studies have demonstrated that it can cause airway inflammation and injury. The airway epithelium forms the first barrier of defense against inhaled toxicants, such as PM. Airway epithelial cells clear airways from inhaled irritants and orchestrate the inflammatory response of airways to these irritants by secreting various lipid mediators, growth factors, chemokines, and cytokines. Studies suggest that PM plays an important role in the pathogenesis of chronic airway diseases by impairing mucociliary function, deteriorating epithelial barrier integrity, and inducing the production of inflammatory mediators while modulating the proliferation and death of airway epithelial cells. Furthermore, PM can modulate epithelial plasticity and airway remodeling, which play central roles in asthma and COPD. This review focuses on the effects of PM on airway injury and epithelial plasticity, and the underlying mechanisms involving mucociliary activity, epithelial barrier function, airway inflammation, epithelial-mesenchymal transition, mesenchymal-epithelial transition, and airway remodeling.

## 1 Introduction

Epidemiological evidence suggests a close association between air pollution, resulting from the increased use of liquid petroleum, coal, and gas in transportation, industry, and domestic settings, and pulmonary mortality and morbidity. According to the World Health Organization (WHO), 99% of the global population breathes polluted air, and low- and middle-income countries are at a greater risk of suffering from the highest exposure (1). Air pollution is estimated to cause more than seven million deaths globally every year (2).

Studies have demonstrated that particulate matter (PM) pollution leads to increased prevalence, emergency room visits, hospitalization, and mortality due to asthma, chronic obstructive pulmonary disease (COPD), lower respiratory tract infections, and lung cancer (3–5). PM is a complex mixture of solid and liquid airborne particles that includes an inert carbonaceous core covered by multiple layers of chemicals, such as sulfates, nitrates, ammonia, sodium chloride, mineral dust, metals, and water (2, 6, 7). Diesel exhaust particles (DEP), which are combusted from liquid petroleum and gas in transport and manufacturing industries, constitute an important fraction of PM pollution (8). PM larger than 10µm are mostly filtered in the nose or throat, while those ≤10µm can be deposited in larger airways. PM ≤2.5µm (PM_2.5_) and ultrafine particles (UFP) (diameter ≤0.1µm) can penetrate deeper into the lung to terminal bronchioles even into the alveoli (9).

The airway epithelium is the first barrier against inhaled toxins and particles. They are formed by polarized epithelial cells that adhere to specialized intercellular connections such as tight junctions and intercellular adhesion molecules (10). The major cell types in the lung epithelium include ciliated, undifferentiated columnar, secretory, and basal cells in the large airways and a similar composition in the small airways, with secretory cells changed to club cells. Bronchiolar and alveolar epithelia consist of type I and II epithelial cells (11). In addition to their barrier functions, airway epithelial cells play an important role in mucociliary clearance of the airways and secrete lipid mediators, growth factors, chemokines, and cytokines as requisites for their metabolic functions (12).

Mechanistic studies have demonstrated that PM, including DEP, can impair ciliary function, induce epithelial permeability, and lead to inflammatory changes, while modulating the proliferation and death of airway epithelial cells (11, 13–16). PM can also affect airway epithelial plasticity and the ability of epithelial cells to reversibly change their phenotype, which has been the focus of extensive research. Epithelial plasticity usually denotes to epithelial-mesenchymal transition (EMT) and mesenchymal-epithelial transition (MET), which reflect the conversion between epithelial and mesenchymal phenotypes. The EMT and MET are affected by injury, inflammation, and repair (17, 18). Urban-like PM can cause EMT- related changes, such as epithelial cell morphology changes, deteriorated intercellular connections, and increased α-smooth muscle actin (α-SMA) and collagen I production in airway epithelial cells (19, 20). Furthermore, DEP were shown to downregulate WNT/β-catenin signaling that is important for epithelial repair in lung organoids (21). Studies of the upper airways have also reported that DEP can cause nasal epithelial disruption (22).

Airway and lung remodeling include the main structural changes observed in chronic respiratory diseases, such as COPD, asthma (23). PM leads to airway remodeling through airway inflammation and injury, and abnormal repair of the airway epithelium (24).

In this review, we focus on the direct and indirect effects of PM on mucociliary activity, airway epithelial barrier function, airway inflammation, epithelial plasticity, and airway remodeling, and discuss the underlying mechanisms.

## 2 Impact of PM on mucociliary function

Air pollutants, including PM, cause mucociliary dysfunction, deteriorate epithelial barrier integrity, and lead to cellular inflammation in the airways. Ciliated epithelial cells and secretory cells, club cells, and goblet cells of the airway epithelium are important for the mucociliary clearance of airways; disorders, defects, and the dysfunction of airway secretion and ciliary activity are associated with severe mortal diseases such as primary ciliary dyskinesia and cystic fibrosis (25). Mucociliary clearance plays an important role in maintaining airway homeostasis, and dysfunction of the airway epithelium occurs in chronic airway diseases such as asthma and COPD (24). Furthermore, studies have reported an increase in mucus production and number of goblet cells in COPD airways (26).

Airway secretory cells secrete serous and mucus containing water, ions, various macromolecules, antimicrobials including defensin and lysosomes, and antiproteases (27, 28), which cover the surface of the airways (28). Mucin glycoproteins (MUC5B and MUC5AC) are the predominant mucus products in human airways; MUC5AC is produced by superficial goblet cells, whereas MUC5B is predominantly secreted by submucosal glands (29). These secretions protect the airways from inhaled environmental insults such as PM, bacteria, viruses, fungi, and other pathogens (27, 30).

Upon inhalation, PM have detrimental effects on the epithelial function and integrity, leading to airway injury and inflammation. Most of the water-soluble portion of PM, which include greater amounts of monosaccharide anhydrides, methoxyphenols, inorganic particles (potassium sulphates and chlorides), and inorganic ions (K^+^, Na^+^, Ca^2+^, NH_4_^+^, Mg^2+^, Cl^-^, NO_3_, SO_4_^2-^), polycyclic aromatic hydrocarbons (PAHs), and various concentrations of inorganic elements (Ca, Fe, Mg, Zn, Mn, Pb and Cu) would be promptly released into the airway surface following inhalation (7, 31). The insoluble portion of PM including higher concentrations of silica/silicates and titanium oxides, and heavy metals such as Fe, Au, vanadium (V), Cu, and Pb may then start an intracellular signal transduction (31, 32).

Studies have reported that DEP induces the secretion of MUC5AC and MUC5B in lung NCI-H292 cells, which is inhibited by TLR4 knockdown (33). Furthermore, PM_2.5_ induces MUC5AC mRNA expression in BEAS-2B human bronchial epithelial cells (HBECs) (34). Studies on urban PM have found that these particles induce the expression of amphiregulin, a ligand for the epidermal growth factor receptor, leading to mucus hypersecretion in HBECs. This was regulated PI3Kα activation and its downstream AKT and extracellular signal-regulated kinase (ERK) pathways (35). More recently, it was demonstrated that PM_2.5_ induced MUC5AC upregulation in human nasal epithelial cells, which forms the initial step of the defense against inhaled irritants and particles in the upper airways. This effect is also blocked by inhibitors of epidermal growth factor receptor (EGFR) and PI3K inhibitors, suggesting that the EGFR-PI3K pathway modulates PM_2.5_-induced MUC5AC expression (36).

**Figure 1:**
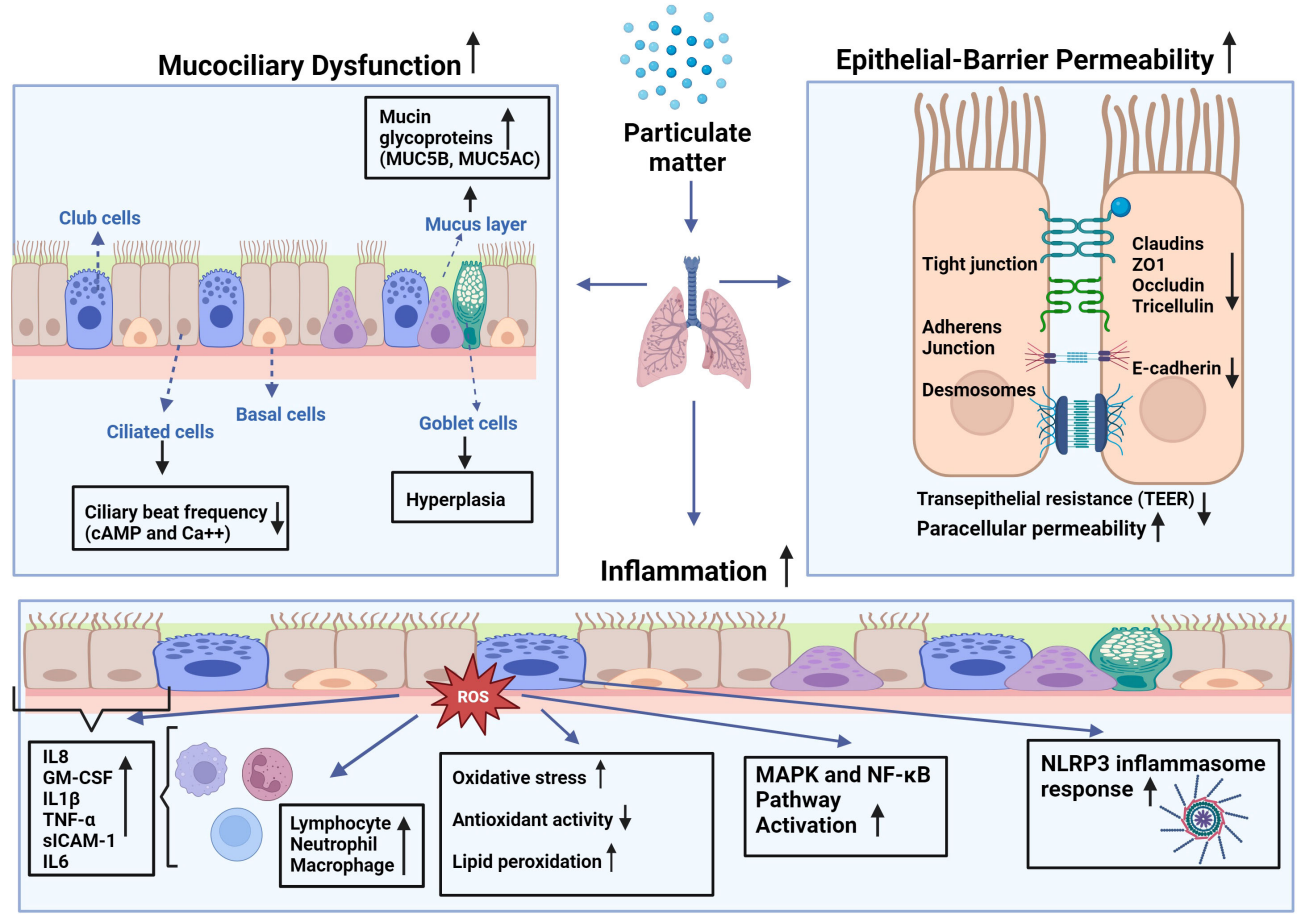
Effects of particulate matter (PM) on mucociliary function, epithelial barrier integrity, and inflammatory changes in airways: PM leads to increased production of reactive oxygen species (ROS) leading to higher oxidative stress in the cell, as well as lipid peroxidation and diminished antioxidant capacity. This imbalance causes augmented expression of inflammatory mediators such as interleukin (IL)-1β, -6, -8, tumor necrosis factor (TNF)-α, granulocyte macrophage-colony stimulating factor (GM-CSF), and soluble intercellular adhesion molecule (sICAM)-1 by epithelial cells and inflammatory cells such as macrophages, neutrophils, and lymphocytes at the site of the inflammation. Increased inflammation may attract more inflammatory cells and lead to increased cytokine production that may cause activation of mitogen-activated protein kinase (MAPK), and nuclear factor-kappa B (NF-κB) pathways, and nod-like receptor pyrin domain-containing 3 (NLRP3) inflammasome response.

Coordinated ciliary motion is important for the clearance of inhaled particles and irritants trapped in the mucus from the airways (11), and substances in the mucus are expelled with the help of ciliary transport or coughing (13). Studies have demonstrated that PM (i.e., DEP) can decrease the ciliary beat frequency (CBF) of primary HBECs (14, 15). Although the underlying mechanisms are not clear, it has been suggested that this could occur through changes in the levels of cyclic adenosine monophosphate (cAMP) and intracellular Ca++, which are known to modulate ciliary movement (37). PM_2.5_ exposure significantly aggravated ciliary deterioration and increased goblet cell hyperplasia in rabbits with chronic rhinosinusitis (38). Taken together, these studies suggest that PM plays a role in the pathogenesis of chronic airway diseases by causing mucociliary dysfunction in the airway epithelium (**Figure 1**).

## 3 Epithelial barrier integrity and effects of PM

The epithelial integrity and barrier function of the airway epithelium are important in the defense of the airways against inhaled insults (**Figure 1**). The airway epithelium forms a selective permeable barrier between the external environment and the underlying tissue. Intercellular connections including tight junction proteins such as zonula occludens (ZO)-1, occludin, claudin-1, and E-cadherin, desmosomes, and intercellular adhesion molecules enable the epithelial barrier to fulfil its protecting role against environmental toxicants (24, 39). The intact epithelium is crucial for the maintenance of tissue homeostasis in protecting the airways and lungs from inhaled irritants, air pollutants, and allergens, and it has been demonstrated that the epithelial barrier deteriorates in chronic airway diseases, such as COPD, asthma, allergic rhinitis, and chronic rhinosinusitis (24, 40–42). Akdis proposed an “epithelial barrier hypothesis”, suggesting that increased environmental toxicants and pollutants associated with industrialization, urbanization, and modern life lead to an increase in allergic, autoimmune, and chronic airway diseases by damaging epithelial barrier integrity (41).

Indeed, studies have demonstrated that exposure to PM can lead to increased epithelial permeability of human nasal epithelial cells, as indicated by increased paracellular permeability and decreased transepithelial electrical resistance (43). This was associated with the downregulation of the tight junction proteins ZO-1, occludin, and claudin‐1. Interestingly, pretreatment of cells with N-acetyl-L-cysteine (NAC) decreased PM_2.5_‐mediated reactive oxygen species (ROS) generation in nasal epithelial cells, preventing barrier dysfunction. Similarly, ambient PM_2.5_ decreased transepithelial electrical resistance and increased paracellular permeability of primary human nasal epithelial cell cultures, which was accompanied by decreased expression of claudin-1, occludin, and ZO-1 proteins and increased inflammatory cytokines (44). Furthermore, urban PM increases ROS production and decreases the expression of tight junction proteins ZO-1, occludin, claudin-1, and E-cadherin, which are associated with an increase in p-Akt, p-p38, and p65 expression in primary human nasal epithelial cells. These effects were reversed by pretreatment with NAC and an Akt inhibitor (39). A DEP study also found that these particles increased the permeability of primary human nasal epithelial cells in a time- and dose-dependent manner, which was associated with a decrease in the expression of occludin, ZO-1, claudin-1, and E-cadherin (45). DEP also decreases transepithelial electrical resistance and increases permeability to dextran in HBECs, which is associated with a reduction in the tight junction membrane protein tricellulin (46). Studies on a human alveolar epithelial cell line (A549) and primary rat alveolar epithelial cells demonstrated that both PM_10_ and DEP led to increased epithelial permeability and disrupted tight junctions while inducing occludin internalization from the plasma membrane into the endosomal compartments and dissociation of occludin from ZO-1. This is prevented by increased expression of the antioxidant enzymes superoxide dismutase (SOD) and catalase (47).

Although evidence from model epithelial monolayers *in vitro* is considerable, there is relatively little evidence regarding the effects of inhaled PM on airway epithelial barrier integrity *in vivo*. Several studies have suggested that the epithelial barrier is compromised in rodents exposed to various forms of PM (48–50). For example, intratracheal instillation of PM_10_ led to increased epithelial permeability in rat lungs, as indicated by enhanced total protein levels in bronchoalveolar lavage (BAL) fluid, which was associated with cell toxicity and inflammation (48). Similarly, ambient PM elevated the BAL protein content in the lungs of mice, which was more prominent in asthmatic mice (49). In a mouse study of ambient PM_2.5_, these particles reduced the expression of E-cadherin in the lung tissue, which was linked to an increase in inflammatory cytokines in the BAL fluid (50). Furthermore, aerosolized DEP causes a significant reduction in the mRNA and protein levels of lung tricellulin 2 (46).

## 4 Inflammatory effects of PM on airways

The vast majority of the literature suggests increases in inflammatory changes in the airways and lungs following PM exposure (51) (**Figure 1**). Studies on primary HBECs have demonstrated DEP-induced release of inflammatory mediators such as interleukin (IL)-8, granulocyte-macrophage colony stimulating factor (GM-CSF), normal T-cell expressed and secreted (RANTES), and soluble intercellular adhesion molecule-1 (sICAM-1) (14, 15). The release from HBECs of allergic asthma patients was greater than that from healthy subjects, suggesting that asthmatic airway epithelial cells are more susceptible to the inflammatory effects of DEP (14). Studies of HBECs of COPD patients reported that repeated exposure to PM with an aerodynamic diameter of 4µM (PM_4_) led to increased release of IL-1, IL-8, tumor necrosis factor (TNF)-α and GM-CSF, and that these cells had a decreased capacity of metabolizing organic chemicals from particles comparing to those cells of healthy subjects (52, 53).

Innate lymphoid cells (ILCs) in the lungs play a role in air pollution-induced inflammatory changes. Indeed, exposure to PM and DEP caused reductions in interferon gamma (IFN-γ) production and type 1 ILC toxicity. These particles also induce the release of IL-5 and IL-13, which play an important role in allergic airway diseases such as asthma, while stimulating type 2 ILCs to produce excess IL-5 and IL-13, leading to airway hyperresponsiveness (54). Previous studies have reported that DEP induces the production of IgE antibodies from B cells, an important marker of allergic diseases (55, 56). Studies on PM have shown that urban fine (PM_2.5–0.2_) and coarse (PM_10–2.5_) particulates could induce the production of nitric oxide (NO), IL-6, and tumor necrosis factor (TNF)-α from mouse macrophages, and that the insoluble fractions of PM were more potent than the soluble PM fractions, suggesting that components adsorbed on PM play a role in their toxicity (57).

Studies of asthmatic murine models demonstrated that PM exposure exacerbated the allergic response by unbalancing T cell response causing airway hyperreactivity together with increased levels of immune cells such as eosinophils, macrophages, and of inflammatory mediators including IgE, IL-4, IL-5, IL-13 and decreases in IFN-γ (58, 59). However, the Th17 response can be enhanced by air pollution. For instance, it has been shown that IL-17 secretion was increased in epithelial cells of severe asthmatics following DEP exposure (60), and that pre-treatment with NAC, a scavenger of ROS and an inhibitor of nuclear factor (NF)-ĸB significantly decreased DEP-induced IL-17A mRNA expression (60). Furthermore, UFPs increase the expression of IL-8, IL-33, and thymic stromal lymphopoietin (TSLP) in airway epithelial cells of patients with severe asthma (61).

In a rat model of COPD, PM_2.5_ exposure led to a decline in lung function and histopathological changes. PM_2.5_ also induced lung inflammation as indicated by increased neutrophils and eosinophils, and inflammatory cytokines such as IL-1β, GM-CSF, and IL-4 in BAL fluid of rats. These changes are accompanied by decreased antioxidant activity and increased lipid peroxidation, suggesting the involvement of inflammatory mechanisms in oxidative stress (62). Similarly, PM_2.5_ led to pulmonary inflammation, decreased lung function, the development of emphysema, increased expression of IL-6 and IL-8, matrix metalloproteinase (MMP)9, MMP12, and transforming growth factor (TGF)-β1 protein in the lungs of COPD mice. Concomitantly, PM_2.5_ increased releases of IL-6 and IL-8, and the expression of MMP9, MMP12, and TGF-β in HBECs. Interestingly, PM_2.5_, further induces inflammatory changes caused by cigarette smoke, suggesting that PM can interact with cigarette smoke during the development and progression of COPD (63). Rui et al. (64) exposed COPD rats to motor vehicle exhaust containing PM_1-10_, finding that the levels of total cell numbers, neutrophils, macrophages, lymphocytes, IL-6, and TNF-α increased in BALF (64). Recently, it was shown that repeated exposure to DEP for eight weeks leads to COPD-like inflammatory changes, including fibrosis, increased total wall area, goblet cell hyperplasia, and increased levels of macrophages in the small airways of rats (65).

It has been demonstrated that both soluble and insoluble fractions of PM_2.5_ cause the production of ROS in water and methanol *in vitro*, and that the insoluble fractions of these PMs are more potent (57, 66). Similarly, DEP, organic extracts of DEP, and polyaromatic hydrocarbons lead to increased levels of ROS in both bronchial and nasal epithelial cells (67), and PM_2.5_ induced ROS in macrophages (68). Increased levels of ROS intracellularly cause oxidative stress, which leads to the activation of mitogen-activated protein kinase (MAPK) pathway activation resulting in the induction of the transcription factors such as nuclear factor kappa B (NF-ĸB) and activator protein (AP)-1 (16, 53). Oxidative stress can also cause DNA and protein damage in cells by activating the nucleotide-binding domain (NOD)-like receptor protein 3 (NLRP3) inflammasome. In an alternative pathway, PM can bind to EGFR on the membrane that may also activate the MAPK pathway together with PI3K/AKT signaling that results in NF-ĸB activation (69). The stimulation of these pathways induces the production of proinflammatory cytokines such as IL-1β, IL-6, IL-8, GM-CSF, and TNF-α. These cytokines can activate innate immune cells such as macrophages, neutrophils, and dendritic cells, which can lead to an inflammatory response (70).

In the innate system, toll-like receptors (TLRs) act as immune sensors and recognize pathogen-associated molecular patterns (PAMPs) of microorganisms and other toxicant stimuli. It has been reported that PM can stimulate airway epithelial cells through TLR2 and TLR4 pathways (70). PM constituents such as PAH can also stimulate cells through aryl hydrocarbon receptors (AhR), which in turn activate proinflammatory intracellular signaling pathways including NF-κB and MAPK pathways (70). PM_2.5_ have also been shown to reduce levels of miRNA331 through ROS/PI3K/AKT pathway, which leads to NF-κB stimulation in human airway epithelial cells resulting in sustained inflammation (71).

Under physiological conditions, immune cells, such as macrophages and neutrophils, engulf exogenous PM and degrade it by phagocytosis through a respiratory burst, which leads to the production of ROS. The mitochondria, which are controlled by cellular and mitochondrial antioxidants, are the main sources of cellular ROS. This organelle is also a target for oxidative damage. Studies have shown that mitochondrial dysfunction due to PM exposure leads to increased oxidative stress and cytotoxic responses, such as apoptosis and necrosis. More recently, we demonstrated that DEP induces the expression of oxidative stress-related genes such as GCLC and cytochrome P4501B1 (CYP1B1) in HBECs (72). Increased mitochondrial ROS production due to PM may cause inflammasome activation (73). Furthermore, some studies have suggested that PM causes epigenetic changes by inducing DNA methylation (74).

## 5 Epithelial plasticity and impact of PM

### 5.1 Principles of epithelial plasticity

The airway epithelium provides a luminal barrier surrounding the airways that carries gases to the alveoli. They are responsible for sensing the environment, secreting, regenerating, and repelling infections, processing toxins, and removing debris from the body (28, 75). The main cell types in the large airways include ciliated, undifferentiated columnar, secretory, and basal cells, whereas in the small airways, secretory cells are replaced by club cells. In the alveolar area, type 1 cells are responsible for gas exchange, and alveolar type 2 cells, which produce surfactants that prevent alveolar collapse and exhibit stem/progenitor cell characteristics, form the bronchiolar and alveolar epithelia (11, 75). Nevertheless, in addition to earlier histological findings, single-cell RNA sequencing (scRNA-seq) data have recently shown a great deal of cellular heterogeneity in the airway epithelium and suggested the existence of unique and/or uncommon cell (sub)types. Some cell types such as ionocytes, neuroendocrine cells, tuft cells, deuterosomal cells, brush cells, M cells, and variant club cells have also been characterized in various studies (76–83). The distribution and proportion of these cell types vary along the proximal-distal axis of the airways to meet local requirements for optimal respiratory function, and it has been reported that this can change in respiratory diseases such as asthma and COPD (84, 85).

Epithelial plasticity is defined as the ability of epithelial cells to reversibly change phenotype and undergo lineage transformations that are not characteristic of steady-state tissue maintenance; it usually indicates EMT and MET, which reflect the conversion between epithelial and mesenchymal phenotypes (17, 86). A terminally differentiated epithelial cell can turn into a progenitor/stem cell, which can produce a new lineage of cells to replace the missing cells in the airway epithelium under normal homeostatic conditions and can maintain its numerical stability in the airway epithelium by self-renewal, which can lead to wound healing and tissue regeneration (17). Depending on the type and severity of the damaging factor, these cells may cause an increase in the population of different cell types, such as mucus-producing cells, secretory cells, or ciliated cells of the same lineage in the airway epithelial cells (75). When the severity of damage increases, differentiated mature cells can transdifferentiate from a distinct lineage to a differentiated cell type (75). Airway epithelial progenitor cells perpetually survey airway homeostasis to sustain a fully differentiated airway epithelium. Basal cells are the primary sources of proximal airway progenitors. These cells are characterized by the expression of transformation-related protein 63 (TP63), cytokeratin (CKs) 5, 6, 8, 13, and 14, and the nerve growth factor receptor (NGFR). Based on these markers, five distinct subtypes of basal cells were identified. Basal cells expressing TP63+CK5+ were identified as quiescent progenitor cells, whereas TP63+CK5+CK14+ and TP63-CK8+ basal cells were identified as proliferative parabasal cells. TP63+CK5-CK14+ cells are classified as hillock basal cells or club cells. Finally, TP63-CK6+CK13+CK14+vimentin+ cells have been reported to function as motile basal cells and play a role in the formation of provisional barriers (75, 87). Recent studies have suggested that basal progenitor cell exhaustion, which is associated with lung function decline, may occur in chronic airway diseases such as COPD (88). More recently, we demonstrated that the levels of progenitor/stem cells expressing CK5, CK14, and p63 markers were decreased in primary bronchial cells cultured from patients with COPD, which correlated with decreased lung function and increased cigarette smoke exposure (89).

Basal cells are the primary source of ciliated cells in large airways (90). In contrast, variant club cells, previously known solely for their ability to secrete and detoxify environmental toxins functions (91), possess the capacity for self-renewal and differentiation into ciliated cells in small airways, particularly in regions devoid of basal cells. In the distal airways and alveoli, type 2 pneumocytes maintain their homeostatic state such that the airways can sustain their structural integrity and function continuously and dynamically (11, 12, 92, 93). These cells renew themselves in response to intercellular signal changes and undergo advanced differentiation into cells or cells responsible for regeneration (93–95).

The differentiation of basal cells into ciliated or secretory cells is believed to be contingent on the balance between Notch 1, Notch 2, and Notch 3 (96). During epithelial homeostasis, these progenitor/stem cells are relatively quiescent owing to the slow turnover of the intact airway epithelium. However, they are also activated in cases of injury (97). Acute injury induces EMT in cells adjacent to the injured area, which then migrate and cover the injured area, providing a temporary patch. In the second step of repair, progenitor/stem cells migrate to and proliferate in the injured areas. In the third step of repair, proliferating cells polarize and undergo redifferentiation to form an intact airway epithelium with normal structure and function. This is a functional endogenous regeneration and repair process (97). However, in the event of severe damage, they acquire danger-associated phenotypes (i.e., increased motility, cytoskeletal rearrangements, and accumulation of extracellular matrix components) to enable a rapid response and subsequent remodeling of a fully differentiated epithelium (98, 99). This leads to reversible (unjamming epithelial transition/immediate EMT) or irreversible EMT and airways (100–102).

During embryogenesis, both EMT and MET are critical for the migration and organization of various cell types into tissues and organs. Under normal homeostatic conditions, these mechanisms participate in complex functional regeneration of the epithelium. However, when dysregulated, both EMT and MET play roles in the migration, invasion, and secondary tumor formation processes under conditions of severe damage to the epithelium, cancer, and fibrosis (103, 104). In particular, following EMT activation in primary tumors, cells increase their capacity to migrate and invade their original tissue, thereby facilitating their dissemination to secondary tissues or organs via the bloodstream. Here, the activation of MET allows mesenchymal-like cells to acquire epithelial characteristics, proliferate, and stimulate angiogenesis, contributing to the formation of secondary tumor tissue (105–107). During EMT, airway epithelial cells lose their characteristic apical-basal polarity, migrate away from the epithelial sheet, and gain mesenchymal characteristics such as front-to-rear polarity, increased motility, and the ability to invade surrounding tissues (100, 105). The inappropriate activation of these processes may contribute to the progression of malignant diseases. Several transcription factors, including the Snail family, zinc-finger E-box-binding (ZEB) family, and basic helix-loop-helix family, are involved in the EMT process (105). TGF-β Wnt/B-catenin, Notch, and connective tissue growth factor (CTGF) pathways induce the expression of transcription factors Snail, Slug, Twist, and ZEB1 (108–110). These factors inhibit E-cadherin expression, decrease epithelial cell-cell adhesion and apicobasal polarity, increase the migration capacity of the resulting mesenchymal cells, upregulate mesenchymal markers such as N-cadherin and vimentin, and promote the secretion of matrix metalloproteases (111). Furthermore, in the downstream of the TGF-β pathway, the IKB/NF-κB pathway regulates Bromodomain Containing Protein 4 (BRD4) to direct the activation of the Snail family transcriptional repressor (SNAI1-ZEB1) mesenchymal transcription factor module (112). Additionally, BRD4 enhances fibroblast growth factors, extracellular matrix (ECM), and MMPs transcription. TGF-β, through the IKK-NF-κB pathway, triggers a transformation in epithelial cells, prompting them to express functional mesenchymal characteristics that includes the upregulation of α-SMA to support cell division, the induction of intermediate filament vimentin for increased cellular motility, and the promotion of ECM formation and deposition through the expression of collagen type 1A, fibronectin 1, and MMP9 (12, 113).

Epithelial plasticity has been observed in various chronic lung diseases including COPD and asthma (86, 112). Disruption of the airway epithelial barrier is a major factor in the initiation and progression of chronic pulmonary disease. This leads to EMT and airway remodeling. Because of epithelial plasticity, epithelial barrier function is disrupted, fibroblastic growth factors are released, and ECM is remodeled (100, 101).

### 5.2 Effect of PM on epithelial plasticity

**Figure 2:**
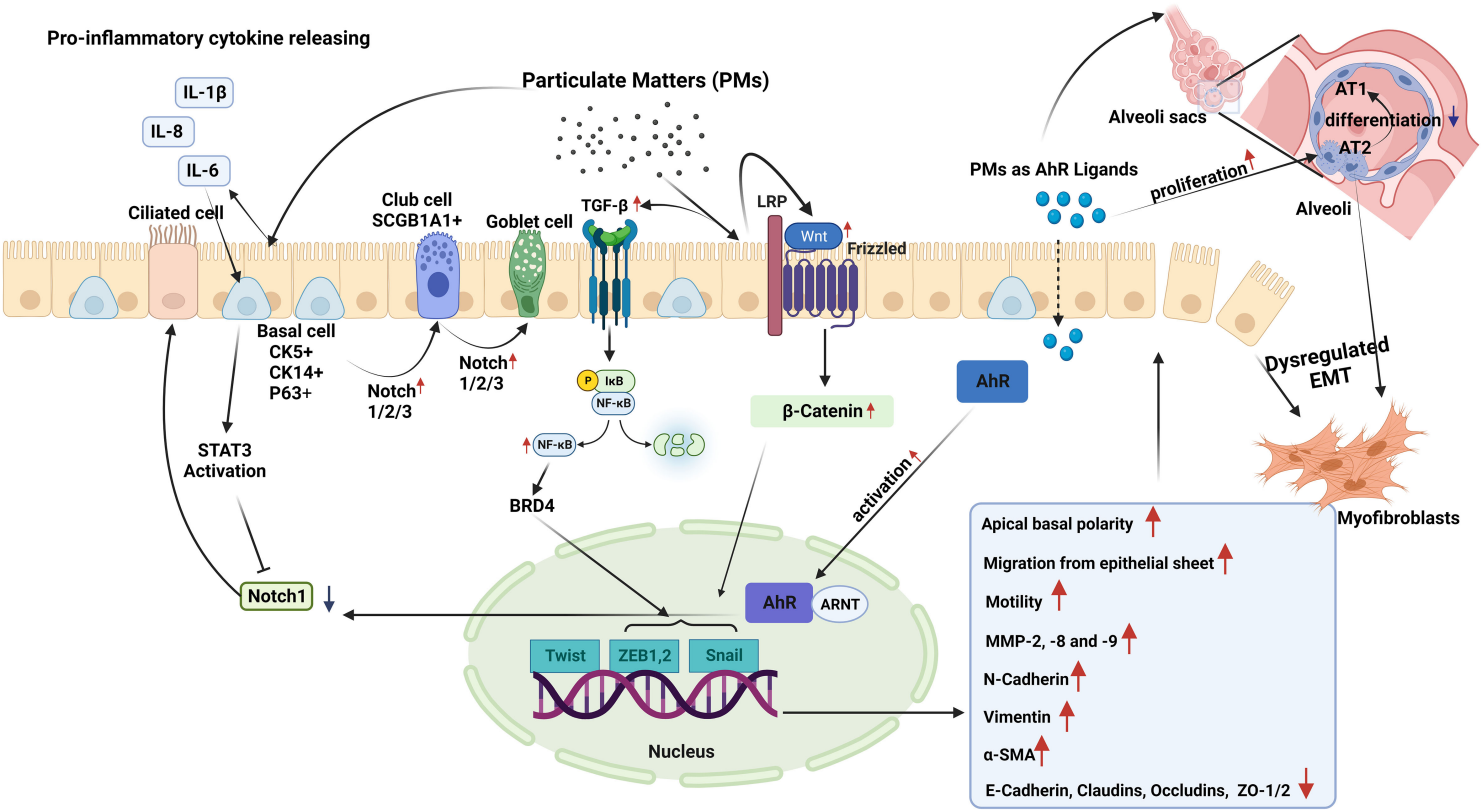
Epithelial plasticity and impact of particulate matter (PM): Exposure to PM in short term, leads to inhibition of Notch1 by stimulating STAT3 and aryl hydrocarbon receptor (AhR) activation in basal epithelial cells. Decreased Notch1 activation leads to differentiation of progenitor/stem cells into ciliated cells. Conversely, longer PM exposure results in increased expression and activation of Notch1/2/3, which induce basal progenitor cells to differentiate into secretory and mucus-producing cells. In areas where there are fewer basal cells, Notch activation stimulates SCG1A1+ club cells to differentiate to goblet cells. Smaller PM ( ≤2.5µM) reaching the alveoli stimulates the proliferation of alveolar type (AT) 2 cells, and induces the differentiation of these cells to myofibroblasts, while preventing their differentiation into AT1 cells. In case of chronic exposure, PM activates the TGF-β-NFκB and Wnt/B-catenin pathways. These pathways activate the Twist, Snail, and Zeb transcription factor module, initiating a process called dysregulated epithelial-mesenchymal transition (EMT). During EMT, there are noticeable changes in apical-basal polarity, migration of epithelial cells from the epithelial sheet, increases in the expression of MMP-2, 8, and 9. E-cadherin, occludin, claudin and zonula ocludens-1 show a decreasing pattern of change, while mesenchymal cell markers such as N-cadherin, Vimentin, and α-SMA increase.

PM can affect the function and integrity of the epithelium, and it has been suggested that under stress conditions, epithelial cells can modify their form and function within tissues (114). Responding to genotoxic stress from environmental exposures such as PM, epithelial cells proliferate and differentiate to replace lost cells with new and functional ones, indicating that normal repair and regeneration processes are operating (17). However, external stimuli, such as air pollutants, may also drive columnar cells into metaplastic squamous cells, and squamous epithelial cells may undergo metaplastic transformation into columnar and goblet cells (114). Unfortunately, the number of studies investigating the impact of PM on epithelial plasticity is limited, and there is currently no research on the impact of PM on MET in airway diseases (**Figure 2**). As discussed above, most studies on MET are associated with its role in the development of malignant diseases (100, 103, 105–107).

A limited number of studies have suggested that inflammatory cytokines, such as IL-6, which can be induced by PM (115, 116), might impact epithelial plasticity. Indeed, in a study utilizing mouse tracheospheres derived from NGFR-positive basal cells, it was demonstrated that IL-6, through the STAT3 signaling pathway, inhibited Notch1 expression. This inhibition leads to an increase in the differentiation of basal cells into ciliated cells, and a decrease in the number of secretory cells (117).

However, PM may also directly affect airway epithelial plasticity through AhR activation (70). This receptor is a unique cellular chemical sensor and ligand-activated transcription factor (118). Upon ligand binding, AhR translocates to the nucleus and heterodimerizes with the AhR translocator (Arnt), which then enables the transcription of many target genes such as the detoxifying enzymes cytochrome P4501A1 (CYP1A1) and CYP1B1 (70, 119). A recent study used club cell-specific AhR-null mice showed that AhR is required for club cell homeostasis via, in part, targeting Notch1 signaling (119). Notch signaling in the respiratory system is essential for lung development. Furthermore, for the pluripotent epithelial progenitors in the developing lung to differentiate into lineage-restricted progenitors of the conducting airways, Notch signaling is necessary (119).

Modulation of Notch signaling creates populations of ciliated pulmonary neuroendocrine (NE), and epithelial club cells (120). It has been documented that Notch signaling regulates stem cell maintenance and differentiation, cell proliferation, and apoptosis, which is necessary for maintaining the homeostasis of the adult lung following lung injury (120). In a study utilizing three-dimensional airway organoids, exposure to tire wire particles, which are traffic-derived PM_10_, led to a reduction in the expression of SCGB1A1, a club cell marker, and CK5, a basal cell marker (121).

In an *in vivo* study, exposure to PM_2.5_ for one month resulted in increased alveolar type (AT) 2 cell proliferation in mice (122). This resolved six months after the cessation of PM_2.5_ exposure. Additionally, experiments on club cell organoids in mice revealed disruptions in progenitor cell capabilities due to PM_2.5_ exposure. In another study using a COPD mouse model and 3D mouse alveolar organoids, PM_2.5_ disrupted AT2-to-AT1 differentiation by altering signaling pathways, including MAPK signaling, Wnt signaling, and pathways regulating the pluripotency of stem cells (123). Wu et al. (21) investigated the effects of DEP on lung organoids derived from co-cultures of alveolar epithelial progenitors (AT2) and fibroblasts. RNA-sequencing analysis revealed that DEP led to downregulation of WNT/β-catenin signaling that led to decreased alveolar organoid growth by reduced AT2 cell numbers. A recent study using human embryonic stem cells investigated the effects of PM_2.5_ on early fetal lung development and found alterations in lung progenitor cell markers such as NKX2.1, SOX2, and SOX9. These changes negatively affect organoid formation (124). More recently, it was reported that SOX2 preserves airway epithelial cell identity and prevents fate changes in functional alveolar tissue or pathological keratinization following lung injury in mouse lungs (125). Additionally, PM_2.5_ exposure reduced the expression of WNT/β-catenin target genes in differentiating cells, potentially influencing proximal-distal airway specification (124).

In addition to their effects on the modulation of epithelial plasticity, PM exposure and severe damage impair repair mechanisms. For example, DEP were shown to downregulate WNT/β-catenin signaling that was important for epithelial repair in lung organoids (21). Antioxidants like NAC and mitoquinone mesylate (MitoQ) reversed the effects of DEP, while a WNT/β-catenin activator (CHIR99021) restored signaling and promoted organoid growth suggesting that oxidative stress might be involved in DEP-induced cellular effects (21). Previous studies have reported that DEP induces ROS production in both human nasal and HBECs (67). Furthermore, we demonstrated that NAC, a catalytic antioxidant, and the superoxide mimetic AEOL10113 prevented DEP-induced lung epithelial cell cycle progression (16). A study on nasal epithelial cells also showed that upon DEP exposure, these cells displayed EMT characteristics regulated by ZEB2, a member of the ZEB family. Interestingly, co-exposure to DEP and house dust mite allergens synergistically induces epithelial disruption and ZEB2 expression, and inhibition of ZEB2 prevents EMT caused by DEP in mice (22).

PM induces EMT by activating the pathways involved in ROS production and mitochondrial dysfunction (126, 127). Additionally, fine PM exposure disturbs cellular processes, such as autophagy, redox balance, and mitochondrial homeostasis, primarily via activation of the c-Jun N-terminal kinase (JNK) pathway. These disruptions inhibit proliferation and promote EMT in alveolar epithelial cells (128). Furthermore, PM_2.5_ dust has been shown to induce autophagy and EMT in human bronchial epithelial 16HBE cells, suggesting a link between PM exposure and EMT (129). Studies have also highlighted the role of PM in activating TGF-β and PI3K/Akt pathways and promoting cell invasion through EMT in A549 cells (130, 131).

## 6 Airway remodeling in chronic airway diseases and effects of PM

**Figure 3:**
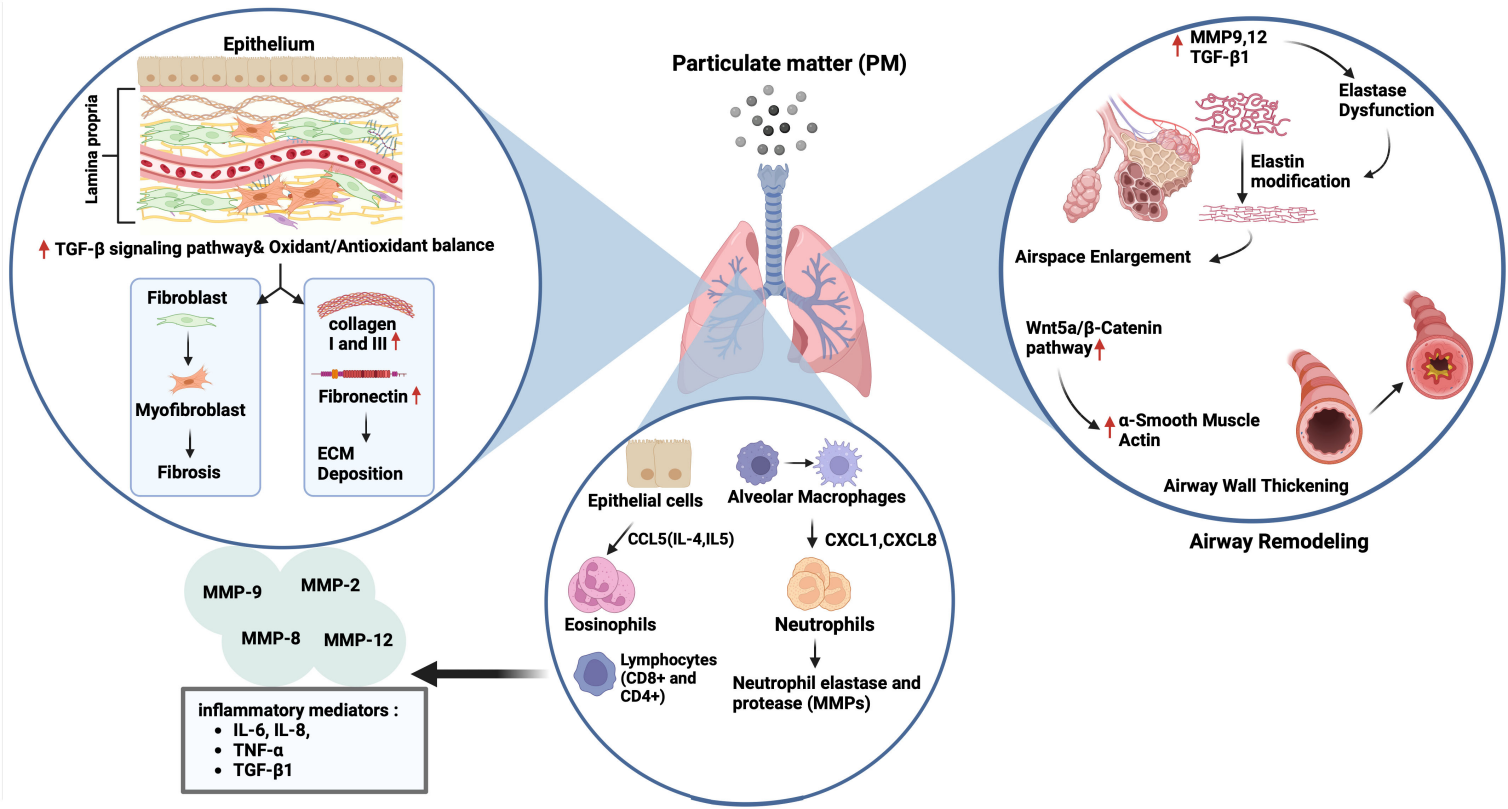
Impact of particulate matter (PM) on airway remodelling. Exposure to PM leads to airspace enlargement and thickening of the airway walls via upregulation of matrix metalloproteinase (MMP) 9,12 and transforming growth factor (TGF)-β1 followed by elastin dysfunction, and activation of the Wnt5a/β-catenin pathway, respectively. On the other hand, the increase and recruitment of immune cells like neutrophils and eosinophils through activation of CCL5, CXCL1, and CXCL6 can occur in the presence of air pollutants, followed by the expression of neutrophil elastase and various MMPs (2, 8, 9 and 12), and inflammatory mediators including interleukin (IL)-6, IL-8, tumour necrosis factor (TNF)-α and TGF-β1. PM exposure also triggers extracellular matrix (ECM) components deposition (collagen I, III and fibronectin) and fibrosis by activation of the TGF-β signalling pathway, and the impairment of oxidant/antioxidant balance.

Tissue remodeling is defined as changes in the quantity, composition, and organization of the tissue structure and is described as a common feature of the repair of tissue damage (132). The remodeling of airways and lungs includes changes in the composition, content, and organization of the cellular and molecular structures of the airway wall in both the small and large airways, and in the lung parenchyma that occurs in chronic respiratory diseases such as COPD, and asthma (23, 133–135). Most tissue remodeling occurs in the ECM components of the airway tissue. The lung ECM is a mixture of various proteins such as collagen, elastin, and glycoproteins, including fibronectin and laminin, which are directly involved in normal lung development (23). Each component plays an important role in the airway and lung parenchyma under both normal and pathological conditions. The ECM in the respiratory system can be modified under pathological conditions such as prolonged exposure to allergens, cigarette smoke, and air pollutants, and a continuous cycle of injury and abnormal repair of the epithelium leads to chronic airway inflammation and remodeling in asthma and COPD (24) (**Figure 3**).

### 6.1 Airway remodeling in asthma and impact of PM

Airway remodeling in asthma is characterized by structural changes in the airway, including loss of epithelial barrier function, goblet cell hyperplasia, deposition of ECM components in the sub-epithelial reticular basement membrane, lamina propria, and submucosa of the airway epithelium, and increased airway smooth muscle (ASM) mass. These changes lead to thickening of the airway wall and narrowing of the airway lumen, causing airflow obstruction (24, 133). Studies have shown that activated fibroblasts in asthma can produce increased levels of collagens, fibronectin, and the profibrotic mediator, TGF-β (136). Furthermore, asthmatic fibroblasts are defective in collagen fiber organization and express less decorin, which is necessary for normal collagen formation (137). In addition to fibroblasts, airway smooth muscle cells have a noticeable impact on the increase in ECM components, such as collagen and fibronectin (135, 138).

Studies have suggested that ambient PM induces airway remodeling in asthma. For example, exposure to PM_2.5_ led to airway inflammation, collagen deposition, and airway remodeling, which were accompanied by a Treg/Th17 imbalance and a decrease in miR-224 in an asthmatic mouse. This was also involved in tissue remodeling factors such as MMPs and TGF-β (62). Similarly, intratracheal instillation of ambient PM_2.5_ caused lung parenchyma inflammation and airway fibrosis in asthmatic rats, which were associated with increased expression of collagen 1, CTGF, IL-6, TGF-β1, Smad3, and p-Smad3. Interestingly, pretreatment of rats with an inhibitor of TGF-β1, and an antagonist of Smad3 reversed the activation of airway fibrosis suggesting that TGF-β1/Smad3 signaling pathway may be responsible for the pathological process of airway fibrosis in asthmatic rats following exposure to PM_2.5_ (139). Multiple intranasal exposures of mice to PM_2.5_ for nine weeks, also led to lung inflammation and type I collagen and hydroxyproline deposition accompanied by increased mitochondrial ROS levels and NADPH oxidase (NOX) activity and decreased total SOD and glutathione (GSH) levels. There was also an increase in the expression of TGFβ1, N-cadherin, and Vimentin, and a decrease in E-cadherin expression suggesting an association with activation of TGFβ1-PI3K/Akt, TGFβ1- NOX and TGFβ1-NLRP3 pathways (140) (**Figure 3**).

### 6.2 COPD, airway remodeling and impact of PM

COPD is characterized by chronic airway inflammation and remodeling, including goblet cell hyperplasia, squamous cell metaplasia, reticular basement fragmentation, changes in the ECM, EMT, altered vascularity, and lamina propria cellularity. Furthermore, small airway fibrosis and thickening and emphysema are observed in patients with COPD (23, 132, 134, 141). One of the main reasons for ECM remodeling in COPD is the inflammatory response, chronic inflammation in COPD is associated with elevated levels of fibroblasts in the airways and an increased number of inflammatory cells, including macrophages, neutrophils, eosinophils, CD4+, and CD8+ T-lymphocytes. Together, these cells secrete MMPs, such as MMP-1, MMP-8, MMP-13, and MMP-12, which degrade ECM molecules and cause destruction and remodeling of the ECM in the small airways and lung parenchyma, leading to airway obstruction (142). Moreover, after analyzing serum samples taken from 20 patients with stable COPD, Zeng et al. found that the levels of IL-6 and procollagen 1 C-terminal peptide (PICP) were elevated in patients with COPDs compared to healthy controls. Interestingly, there was a positive correlation between inflammatory cytokines, such as IL-6 and IL-8, and PICP concentration (142). The authors suggested that increased PICP levels may indicate airway remodeling and that these cytokines may play a role in collagen synthesis (143).

Chronic exposure to tobacco smoke is the main risk factor for pathophysiological changes and remodeling in COPD through MMP2, MMP8, MMP9, and prolyl endopeptidase expression, which can cleave collagen and induce collagen deposition in the terminal bronchioles (24, 132, 133). Other inhaled irritants, including air pollutants such as atmospheric PM, also play a role in the development of the disease. However, studies investigating the impact of air pollution on COPD development and the underlying mechanisms are limited because animal models of COPD present few features of the disease, and the best animal models display limited pathological characteristics of COPD. These models are expensive and time-consuming (4, 144). To compensate for these limitations, various culture models have been established, including 3D organoid cultures, microfluidic cell culture systems, and organ-on-a-chip models, for COPD studies (26, 145). These living cell-organ systems aim to establish complex 3D structures to provide an organ-like *in vivo* system representing the anatomical region from which they originate. These models have been successfully applied in recent years (26, 53, 145).

Studies using animal models of COPD have suggested that PM exposure induces tissue remodeling (62, 63, 146). For example, PM_2.5_ led to increases in expression of Collagen 1, Collagen 3, and the profibrotic cytokine α-SMA, and TGF-β1 in lungs of rats with COPD via disrupting oxidant/antioxidant balance (34). Furthermore, Zou et al. (146) reported that PM_2.5_ caused emphysema, airway wall thickening, and increased smooth muscle layer thickness, and overexpression of the Wnt5a/β-catenin pathway in mice. PM_2.5_ also increased the mRNA expression of the Wnt5a, β-Catenin, TGF-β1, cyclin D1, and c-myc mRNAs in human bronchial smooth muscle cells (HBSMCs). Protein expression of proliferating cell nuclear antigen (PCNA), α-SMA, Wnt5a, β-Catenin, platelet-derived growth factor receptor (PDGFR) β, and tenascin C was induced by PM_2.5_ in these cells that were inhibited by BOX5, an antagonist of Wnt5a. Together, the authors suggested that PM_2.5_ could lead to airway remodeling by inducing HBSMC proliferation via the Wnt5a/β-Catenin signaling pathway both *in vivo* and *in vitro*. Similarly, chronic prolonged exposure to ambient PM_2.5_ led to pulmonary inflammation, impaired lung function, development of emphysematous lesions, and airway wall remodeling that was indicated by airspace enlargement, increased expression of IL-6 and IL-8, MMP9, MMP12, and TGF-β1 proteins in lungs of COPD mice model. Similarly, PM_2.5_ increased releases of IL-6 and IL-8, and the expression of MMP9, MMP12 and TGF-β, fibronectin, collagen I and III in HBECs (63). In a recent study, the authors established a rat model of COPD by exposure to motor vehicle exhaust and found that rats developed lung function decline, lung inflammation, emphysema-like alveolar enlargement, and airway remodeling (64) (**Figure 3**). Nevertheless, their findings may not solely reflect the effects of PM, as motor vehicle exhaust also contains pollutant gases, such as CO, NO, and SO_2_ together with PM of different diameters (147).

## 7 Discussion

Air pollution is the major cause of respiratory morbidity and morbidity worldwide (2). PM, a complex mixture of solid and liquid airborne particles, plays an important role in the pathogenesis of chronic airway diseases, such as asthma and COPD (4). PM size is an important determinant of toxicity; the smaller the particle diameter, the more toxic it is (7). Airway epithelial cells form the first barrier of defense against inhaled toxicants such as PM (10), play an important role in mucociliary clearance of the airways, and orchestrate the inflammatory response of airways to inhaled toxicants by secreting lipid mediators, growth factors, chemokines, and cytokines (11). Studies suggest that PM leads to airway pathologies by impairing mucociliary function, deteriorating epithelial barrier integrity, and inducing inflammatory changes while impacting epithelial plasticity and airway remodeling (53).

Most PM studies on epithelial cell models have been performed using *in vitro* human and animal cell lines, which may not adequately represent the physiological and biochemical properties of primary cells or *in vivo* conditions. Many studies have reported challenges in determining the common effects of PM owing to the use of different cell lines and varying passage numbers. The number of studies utilizing primary cells obtained from healthy individuals or patients with chronic airway diseases such as asthma and COPD is limited. Additionally, heterogeneity in PM extraction methods and the loss of true positive controls are problematic (53, 148). Animal models may have limitations such as high cost and inadequate representation of disease models (4, 144). Recent studies have used organoid (3D) cell culture systems that are embedded in a matrix and present tissue-like structures containing tissue-specific cells. These models also provide longer exposure times, which are usually not possible in 2D cell culture systems (26, 53, 92, 145).

Mucociliary clearance plays an important role in maintaining airway homeostasis and clearing the airways from inhaled irritants and toxicants. Mucociliary dysfunction of the airway epithelium occurs in chronic airway diseases, such as asthma and COPD (24). Studies have demonstrated that PM induces mucus secretion through mechanisms involving the expression of MUC5AC, MUC5B, amphiregulin, and the activation of the AKT, ERK, EGFR, and PI3K pathways in airway epithelial cell systems (33–36) (**Figure 1**). However, it is unclear whether airway epithelial cells of patients with asthma or COPD are more susceptible to the effects of PM on mucus production. On the other hand, a limited number of studies have found that DEP decreases CBF in primary HBECs cultured from healthy and asthmatic patients (14, 15). To the best of our knowledge, no study has investigated the impact of PM on ciliary activity in airway epithelial cells in COPDs. Furthermore, it would be useful to study the roles of intracellular cAMP and Ca++, which modulate ciliary activity, in PM-induced CBF inhibition (37).

Epithelial integrity is crucial to prevent inhaled irritants from penetrating the subepithelium and passing deeper into the lung parenchyma to exert deleterious effects. Various studies from epithelial monolayers reported that PM led to increases in permeability of nasal (43), bronchial (46), and alveolar epithelial cell cultures (47) that was associated with disrupted tight junctions, downregulation of tight junction proteins such as ZO-1, occludin, claudin‐1, and E-cadherin (**Figure 1**). However, studies investigating the effects of PM on airway epithelial barrier integrity in *in vivo* models are lacking. Several animal studies have suggested increased permeability in the lungs, as indicated by increases in total protein levels in BALF (49) and decreased expression of E-cadherin (50) and tricellulin 2 (46) in lung tissue. However, studies using airway microfluidic and 3D organoid models may overcome this problem and provide better insights into the *in vivo* effects of air pollutants on epithelial integrity (53).

There is relatively more evidence of the inflammatory effects of PM on the airway epithelium. It has been shown that PM induces the gene and protein expression of various inflammatory mediators from airway epithelial cells that play an important role in the pathogenesis of chronic airway diseases, including asthma and COPD (14, 52). Mechanistic studies suggest that PMs exert their effects through the production of ROS at cellular levels (67, 68), and that the cellular pathways involving oxidative stress are activated, since antioxidants such as NAC and inhibitors of NF-κB and MAPK pathway could inhibit these effects (16, 60) (**Figure 1**). Most primary human airway cell studies have been conducted in patients with asthma (14) and allergic rhinitis (55), whereas COPD studies have mostly been conducted in animal models (65). Therefore, further studies on primary cell systems such as organoids are needed to investigate the role of PMs in COPD pathogenesis.

Epithelial plasticity (EMT and MET), plays an important role in embryonic development and tissue responses to damage, including inflammation and repair. Epithelial plasticity plays a key role in homeostatic processes such as wound healing and tissue regeneration. Studies suggest that epithelial plasticity can also be effective in the pathogenesis of chronic lung diseases, such as COPD and asthma (86, 112). A few studies have suggested that PM affects airway epithelial plasticity by modulating airway injury, inflammation, and repair (148). Although the impact of PM on EMT has been investigated to some extent in recent studies, to the best of our knowledge, no study has investigated the effects of PM on MET, which is critical for wound healing, tissue repair, and regeneration (17).

It has been suggested that acute exposure to PMs, through increased production of cytokines such as IL-6 (115), STAT3 activation (117) and AhR activation (70), causes Notch1 inhibition (70) which leads to the differentiation of progenitor/stem cells into ciliated cells (117). Activation of Notch signaling has also been reported to regulate cell proliferation and apoptosis (120). More recently, exposure to PM_10_ led to a reduction in the gene expression of club and basal cell markers in airway organoids (121). Furthermore, PM_2.5_ led to AT2 cell proliferation in mice, is associated with disruptions in the progenitor cell capabilities of organoids from mouse club cells (122). In a COPD mouse model and 3D mouse alveolar organoids, PM_2.5_ disrupted AT2-to-AT1 differentiation via mechanisms involving the MAPK and Wnt signaling pathways (123). It was also found that downregulation of WNT/β-catenin signaling by DEP led to decreased AT2 cell numbers in alveolar organoids of mice (21). PMs have also been shown to deteriorate mitochondrial function (126, 127, 129) and disturb autophagy, redox balance, and mitochondrial homeostasis, primarily via the activation of the JNK pathway (131). Together, these findings suggest that PM can induce EMT in the airway epithelium while impairing repair mechanisms involving epithelial regeneration (**Figure 2**).

If the EMT induced by PM cannot be stopped, MET cannot be started, and the epithelium is not successfully repaired; this process may lead to airway and lung remodeling, which are typically seen in chronic respiratory diseases such as COPD and asthma. Indeed, studies demonstrated that PM led to collagen deposition, airway remodeling and fibrosis, which were associated with the activation of MMPs and TGF-β pathway, and increased expression of inflammatory and profibrotic mediators in both asthma (62) and COPD (63) models. Intratracheal instillation of PM_2.5_ caused airway fibrosis in asthmatic rats associated with increased expression of collagen 1, CTGF, IL-6, TGF-β1, Smad3, and p-Smad3 that was reversed by inhibiting of TGF-β1, and Smad3 suggesting the role of TGF-β1/Smad3 signaling pathway in asthmatic mice (139). Other studies additionally suggest the role of TGFβ1-PI3K/Akt, TGFβ1- NOX, and TGFβ1-NLRP3 pathways, were associated with increased oxidative stress and decreased antioxidant activity (140). These mechanisms were also reported to be effective in COPD animal models (62). Additionally, chronic exposure to PM_2.5_ caused emphysematous lesions such as airspace enlargement together with increased expression of inflammatory cytokines, MMP9, MMP12, and TGF-β1 in lungs of COPD mice (63, 64).

In conclusion, although various aspects of the detrimental effects of PM on the airways have been investigated, further research is needed to fully understand the effects of PM on the airway epithelium and the underlying mechanisms of epithelial plasticity and remodeling. In particular, the use of 3D culture models and omics techniques (bulk or single cells) may provide a more comprehensive approach for elucidating the role of these particles in chronic airway diseases. Furthermore, the role of agents that target specific pathways and factors involved in PM-induced modulation of epithelial plasticity and remodeling should be investigated to understand their potential role in the prevention and mitigation of the deleterious effects of PM.

## Author contributions

ÖK: Conceptualization, Data curation, Formal analysis, Investigation, Supervision, Writing – original draft, Writing – review & editing. HR: Data curation, Formal analysis, Investigation, Writing – original draft, Writing – review & editing, Visualization. NK: Formal analysis, Investigation, Writing – original draft, Writing – review & editing, Visualization. DM: Formal analysis, Writing – original draft, Writing – review & editing, Visualization. GA: Formal analysis, Writing – original draft, Writing – review & editing, Visualization. JW: Formal analysis, Writing – review & editing. HB: Conceptualization, Data curation, Formal analysis, Funding acquisition, Investigation, Supervision, Writing – original draft, Writing – review & editing.
